# Mechanical stretch and chronotherapeutic techniques for progenitor cell transplantation and biomaterials

**DOI:** 10.1051/bmdcn/2018080314

**Published:** 2018-08-24

**Authors:** Eve Helena Rogers, Vanja Pekovic-Vaughan, John Alan Hunt

**Affiliations:** 1 Institute of Ageing and Chronic Disease, University of Liverpool the William Henry Duncan Building, 6 West Derby Street Liverpool UK L7 8TX; 2 School of Science and Technology, Nottingham Trent University Clifton Campus, College Drive Nottingham UK NG11 8NS

**Keywords:** Progenitor cells, Circadian clock, Mechanical stretch, Cell differentiation

## Abstract

In the body, mesenchymal progenitor cells are subjected to a substantial amount external force from different mechanical stresses, each potentially influences their behaviour and maintenance differentially. Tensile stress, or compression loading are just two of these forces, and here we examine the role of cyclical or dynamic mechanical loading on progenitor cell proliferation and differentiation, as well as on other cellular processes including cell morphology, apoptosis and matrix mineralisation. Moreover, we also examine how mechanical stretch can be used to optimise and ready biomaterials before their implantation, and examine the role of the circadian rhythm, the body’s innate time keeping system, on biomaterial delivery and acceptance. Finally, we also investigate the effect of mechanical stretch on the circadian rhythm of progenitor cells, as research suggests that mechanical stimulation may be sufficient in itself to synchronise the circadian rhythm of human adult progenitor cells alone, and has also been linked to progenitor cell function. If proven correct, this could offer a novel, non-intrusive method by which human adult progenitor cells may be activated or preconditioned, being readied for differentiation, so that they may be more successfully integrated within a host body, thereby improving tissue engineering techniques and the efficacy of cellular therapies.

## 1. Introduction: human mesenchymal progenitor cell response to stretch

Precisely defining and controlling the fabrication of cell/tissue constructs so that they can be successfully delivered and integrated within a patient, is an essential consideration in cellular therapies. Adult progenitor cells are the current first choice, but there are many aspects of their environmental requirements that are still not completely understood. Mesenchymal stem cells (MSCs), which are multipotent progenitor cells [1], are frequently selected in order to replace and restore the function of deteriorated or damaged tissue. This is because of their ability to be pre-differentiated and seeded into biomaterials before their implantation, in order to ensure optimum delivery and integration of a specific cell type. MSCs are advantageous as they have the capacity to differentiate into a number of cell types including, but not necessarily limited to: fat, bone, tendon, muscle, skin, neural, cartilage, dentinogenic, marrow stroma and vascular cell types [2]. Therefore, one of the key goals in the fields of tissue engineering and regenerative medicine is to optimise the conditions for the MSCs, so that differentiation can be predetermined and terminally lineage specific upon providing appropriate stimulation. The most frequently utilised method of cellular MSC differentiation involves adding chemical agents, which can be supplemented into media, and allowing the cells to differentiate slowly over many weeks; ideally up to a month. However, although this is quite practical *in vitro,* it is not the case *in vivo.* Researchers and tissue engineers are therefore striving for a method to optimise the culture conditions so that constructs can be readied *in vitro*, before being implanted *in vivo*, where they should integrate rapidly and start to form the desired target tissue.

One stimulus which may fulfil both requisites is mechanical stimulation. MSCs have been previously shown to be highly mechanosensitive and therefore mechanical stimulation may present the ideal method to non-invasively steer their differentiation, as mechanical forces have the capability of influencing progenitor cell behaviour. In the body, various tissues are exposed to varying amounts of mechanical force, which then influences their formation and functionality; for example, in the body, MSCs found within adipose tissue (ADSCs), will be exposed to vastly different levels of external force compared to those found within the bone marrow (BM-MSCs), teeth (DPSCs), tendon or muscle. This mechanical physiological loading is vital in the maintenance of such tissue; for example, exercise, whereby there is an increase in physical perturbation in terms of load, the magnitude, and the frequency. This results in increases in bone and muscle mass [3], but decreases in physical loads, as experienced in the extreme case of space travel or following injury, that effects physical movement for example, spinal cord damage, tissues like bone and muscle will be lost and decrease [4].

There are many different types of force that may be applied to MSCs *in vivo* and *in vitro.* In the bone marrow alone, MSCs may be subjected to extrinsic stresses such as tension, compression and fluid movement induced shear stress, as well as intrinsic stresses such as substrate, extracellular matrix stiffness, and these are all thought to have individually significant potential and roles on the different differentiation pathways that an MSC may go down. However, the optimum conditions to control and reproducibly define lineage specific differentiation of MSCs remains unknown, and the optimum loading magnitude, duration, frequency and force type for different lineage specific differentiation pathway remain unspecified. In a review by Smith and Reilly (2012), the authors examine how each of following types of force may effect MSC maintenance and differentiation: stretching (tensile stress), hydrostatic pressure or platen abutament (compressive stress), fluid flow (shear stress), ultrasound, high frequency low magnitude displacement (vibration) and direct cell membrane magnetic stimuli, in both 2D and 3D culture systems [5].

There are several proposed methods that may explain how extracellular mechanical stimuli is converted into biochemical signals, which ultimately leads to the cellular changes seen poststimulation. One such mechanotrandsuction mechanism implies cell membrane mechanoreceptors, including integrins, g-protein coupled receptors (GCPRs) and stretch activated ion channels as vital components of this signal transduction. Regarding integrins, it is thought that the mechanical force pulls on an integrin-ligand bond, which is then transferred across the cell membrane and alters the cytoskeletal structure. For stretch activated ion channels and GPCRs, it is theorised that the stretch or external force leads to deformation of the plasma membrane, which results in ion flux into and out of the cell through the receptors [6]. Indeed, when MSCs are strained in the presence of the stretch-activated cation channel (SACC) blocker, gadolinium chloride (GdCl_3_), there is a reduction in the otherwise observed induction of collagen I expression [7], suggesting a role for these channels in the transduction of mechanical stimulation. In the case of fluid flow, it is also thought that the glycocalyx, a GAG-proteoglycan rich layer that surrounds the cell membrane, may create drag force when fluid passes over, which again results in plasma membrane deformation [8, 9]. A final proposed mechanotransuction mechanism, again relevant to fluid flow, insinuates the primary cilium as being a mechanosensor, as they have been shown to bend under fluid flow and contain various signalling receptors [10]. However, this review principally focuses on the effect of mechanical stretch or tensile stress in adult MSCs.

**Fig. 1:**
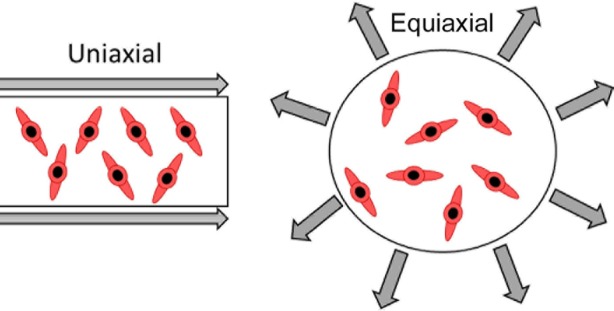
Comparison of uniaxial and equiaxial strain.

*In vitro,* mechanical stretch is usually applied using a mechanical stretch system available “off the shelf” commercially or utilising a custom-built device made to deliver uniaxial mechanical loading at varying frequencies and magnitudes. The application of one such custom system is demonstrated by Kurpinski and Song (2007) [11]. Uniaxial strain is typically selected over eqiaxial strain (Fig. 1) as it is thought to better mimic the type of mechanical strain exhibited by MSCs in the body. For example, research by Park *et al.* (2004) directly compare the effects of uniaxial vs eqiaxial strain in MSCs and find that the different modes induce different responses. Cyclic eqiaxial stretch is here shown to downregulate the smooth muscle differentiation markers SM α-actin and SM-22α, and decreases α-actin in stress fibres. In contrast, cyclic uniaxial strain transiently increases the levels of SM α-actin and SM-22α, suggesting that this method better mimics the type of mechanical strain experienced in MSCs and smooth muscle cells (SMCs) and may promote the differentiation of MSCs into SMCs [12].

As is this case in this study, along with many others, silicone is usually the selected material used as a substrate to study the behaviour of cells under mechanical stimulation. It is selected due to its biological inertness, translucency, compliance and manipulability, and can be coated in fibronectin to allow for good cell adhesion and proliferation. Flexible silicone substrates can also be used to study the effects of mechanical stretch in 3D; research by O’Caerbhaill *et al.* (2007) shows that it can be constructed as a tube to form ‘pseudovessels’, whereby cells can be subjected to a combination of pulsatile flow, radial distension and shear stress. Here, the MSCs are shown to be mechanosensitive, and reorientate parallel to the direction of flow and adapt their morphology in response to the stretch and other forces that they are subjected to [13].

The effect of cyclic uniaxial strain on elastic substrates on the morphology of cultured cells has been studied in great detail. Stretching results in alterations in cell orientation and the cells tend to align perpendicularly to the load axis, in order to lessen the strain on their cell bodies and decrease the stretch of cytoskeletal elements and intercellular filaments [14, 15]. Mechanical stretch can also be investigated in conjunction with the addition of chemical agents, so that their effects can be compared with and without their addition, and with and without stretch. For example, Rashidi *et al.* (2012) combined stretch with growth factor treatment, and found a significant up-regulation of smooth muscle cell specific markers and alignment of cells perpendicular to the strain direction during loading time. They also observed cell elongation and F-actin fibres alignment and reorganisation [16]. The degree of morphological alteration can be seen by these changes in cell orientation and alignment, and both depend on strain amplitude [17], rate and duration [18]. Parankakh *et al.* (2017) sought to investigate the effects of different durations of cyclic stretch on cytoskeletal reorganisation and morphology of human MSCs in a stepwise manner, in order to closely study morphological and phenotypic changes, as the two often occur simultaneously. The researchers found that different durations of stretch did influence the resulting changes observed [19]. Morita *et al.* (2013) also sought to define the optimum conditions for the cyclic stretching of human MSCs. Here, the authors used a 2D inhomogeneous membrane strain field, achieved by removing holes in one side of an elastic chamber, in a commonly used uniaxial stretching device and found the axial strain threshold of hBMSCs was 4.4 ± 0.3 % [20]. Once the optimum parameters for cyclic cellular stretch are defined, this will offer a non-invasive methodology by which human MSCs may be controlled and manipulated, so that they can be optimised for tissue engineering technologies.

## 2. The effect of tensile strain on progenitor cell proliferation and differentiation

### 2.1. Proliferation

The effects that radial distention, or mechanical stretch, can bring about on human adult progenitor cell proliferation have been investigated in a plethora of studies, with each varying the parameters in different ways. For example, in rat MSCs isolated from bone marrow and subjected to cyclical eqiaxial stretch, the OD values of rat MSCs increase in a time-dependent and magnitudedependent manner after being exposed to 2-8% stretch, for within 15-60 min, at a frequency of 1 Hz, suggesting that cell proliferation increases following short term stretch. The expression of c-fos, a regulator of cell proliferation, in these cells is also significantly higher when the cells are stretched (1 Hz, 8% strain and 60 min) compared to static controls; this suggests that mechanical stretch alone could influence cell growth and proliferation [21]. In human cells, fibronectin coated silicone chambers have been utilised to stretch human MSCs, and here it was also documented that the short time application of strain did affect proliferation. Protein coating using fibronectin, however, did not influence MSC proliferation. The authors also looked at the modulation of stretch magnitude, frequency and duration, and found that a frequency of 1 Hz was most effective at stimulating human MSC proliferation. At a frequency of 1 Hz and durations of 15, 30, 60 min, 5% strain was found to significantly increase MSC proliferation. Proliferation was also enhanced at 10% strain, 1 Hz for 15 and 30 min durations, but proliferation decreased at 60 min. At 1 Hz, 15% strain, proliferation was reduced following 15 min durations, but increased following 30 and 60 min durations. Longtime strain applications (12 and 24 h) were found to block proliferation [22]. Collectively, both of these studies highlight how crucial getting strain application conditions optimal are in getting the desired result, and demonstrate that mechanical strain does have the potential to influence MSC proliferation.

The mechanisms that underlie the transduction of mechanical stretch information onto cellular proliferation have been investigated. In a follow up study using the same rat MSCs as mentioned previously, it has been reported that although no change in the expression of total extracellular signal-regulated kinase 1/2 (total ERK1/2) at the protein level was observed, the phosphorylation of ERK1/2 was increased after stretch. When rat MSCs are treated with inhibitors of ERK1/2 activity, there was a suppression of stretch-induced increase in phosphorylated ERK1/2 and mRNA expression of c-fos, along with an abolition of the increase in stretch-induced proliferation, suggesting that ERK1/2 is crucial in the stretch-induced proliferation of rat MSCs [23].

### 2.2. Myogenesis

In a study by Ghazanfari *et al.* (2009), the authors found data that suggested that cyclic strain not only enhanced proliferation, in agreement with the studies above, but also that cyclic strain lead to increases in smooth muscle α-actin, reoriented actin fibres and led to the differentiation of human MSCs into SMCs, without the addition of growth factors [24], implying that mechanical stress can be used to enhance smooth muscle myogenesis [25]. This can also be observed in skeletal muscle myogenesis, which is logical as both foetal and adult skeletal muscle are constantly subjected to biomechanical forces in the body. One study used a 10% uniaxial strain at 1 Hz on human MSCs cultured on collagen-coated silicone substrates and found that, following loading, there was a rearrangement of cells and initiation of myogenic differentiation, as determined by levels of MyoD and MyoG mRNA levels, both of which are key factors in myogenesis, indicating that cyclic strain may be used to differentiate progenitor cells myogenically [26].

Cardiomyocytes are also subject to cyclic strain in the body, as induced by the rhythmic beating of the heart. When rat BM-MSCs were subjected to cyclic strain application, it has been shown that this may be sufficient to induce cardiomyogenic differentiation in itself, as can be confirmed by the induction of cardiomyocyte-related markers [27]. This too suggests that mechanical stimulation could be a novel mechanism to control adult progenitor cell differentiation.

### 2.3. Osteogenesis

Another part of the body in which adult progenitor cells reside and are subjected to varying amounts of mechanical conditioning is in bone; as previously mentioned, BM-MSCs are subjected to external forces such as tension, compression and fluid-induced shear stress. Mechanical stimulation is so crucial in bone formation and maintenance that “distraction osteogenesis” has been created; an active process for bone regeneration using mechanical stimuli. This process has been mimicked experimentally, whereby rat MSCs are subjected to short periods of cyclica mechanical strain (40 min, 2000 microstrains). Following this, it can be observed that mechanical strain promotes ALP activity, as is vital in the initiation of bone formation, and enhances bone marker genes Cbfa1 and Ets-1 expression, showing that mechanical strain may act as a stimulator of osteogenic differentiation [28, 29]. Kearney *et al.* (2010) also document an increased expression of osteogenic markers following cyclic tensile mechanical strain of 2.5% at 0.17 Hz for 1–14 days; the osteogenic markers Cbfαl, collagen type I, osteocalcin, and BMP2 are temporally expressed. However, this strain-induced increase in BMP2 can be reduced by the inhibitors of the kinases, ERK, p38, and PI3 kinase. The authors also found that this long term application of strain reduced the proliferative capacity of MSCs, supporting the notion that although short-term strains may increase proliferation, long-term strains do not appear to [7]. It has also been shown that both osteoblastogenesis and osteoclastogenesis are influenced by mechanical stimulation, showing how far reaching the effects of mechanical load are in bone formation and maintenance [30, 31].

There are many signalling pathways that have been implicated regarding how mechanical stimulation effects osteogenesis, for example, one study shows that the onset of osteogenic differentiation following mechanical stimulation may be dependent on ERK1/2-Runx2 signalling [30]. Another study investigating human BM-MSCs following stretch, reports an induction of FosB, a member of the AP-1 family of transcription factors which regulate osteogenic differentiation and bone formation, in a time-and stretch-dependent manner [32]. The p38MAPK-osterix pathway has also been implicated; intermittent stretching has been found to promote the expression of osterix mRNA, along with ALP, collagen type I and osteocalcin, and the protein levels of osterix and phosphorylated p38MAPK were elevated following stretch. When osterix was silenced, a reduction in the levels of ALP, collagen I and osteocalcin mRNA were also observed, showing that this pathway may have an important role in stretch-induced osteogenesis [33]. A further factor that has been implicated in osteogenic differentiation following tensile strain, without the addition of osteogenic supplements, is BMP-234. Interestingly, even adipose derived MSCs (ADSCs) can be induced to express increased levels of BMP-2 and Runx2 following cyclic tensile strain of 6 hours, underlining the suggestion that cyclic tensile stretch may modulate osteogenic differentiation, *via* the BMP-2 signalling pathway [35].

In direct contrast to the above notion that mechanical stretch results in increased osteogenesis, this does not appear to be the case in all MSCs. MSCs derived from tooth dental pulp (DPSCs) appear to show the opposite, and instead exhibit increased levels of proliferation and decreased osteogenic potential following uniaxial mechanical stretch [36]. Indeed, when human DPSCs were exposed to cyclic tensile stretch, the expression of osteogenic marker genes and proteins including BMP-2, osteocalcin and ALP were reduced, along with the odontogenic marker genes and proteins DSPP, DSP and BSP, suggesting that cyclic tensile stretch inhibits both osteogenic and odontogenic differentiation in DPSCs [37]. The differences in the response of the MSCs depending on their tissue of origin may be due to the fact that DPSCs reside within a unique niche in the body, where they are subjected to extreme mechanical stresses by jaw movement, occlusal forces and hydrostatic pressures, and are one of the few progenitor cell niches to also experience thermal shock and extreme temperature fluctuations, and so are likely to respond differently to mechanical stimulation. Apart from in these unique DPSCs, the effect of mechanical stimulation on the osteogenic differentiation capabilities of MSCs is profound, even having such a strong impact that it forces even adipose derived progenitor cells (ADSCs) to undergo osteogenesis and inhibits their adipogenic differentiation potential, even when cultured in the presence of adipogenic medium [38].

### 2.4. Adipogenesis

The theory that mechanical stimulation inhibits adipogenesis has been widely explored in scientific research. Initially investigated in C2C12 myoblasts, myoblast-to-adipocyte differentiation was found to be significantly inhibited by cyclic mechanical stretch (20% elongation), which was seen alongside an enhanced expression of Wnt10b mRNA. By inhibiting Wnt signalling with a Wnt ligand, sFRP-2, this inhibition of adipogenesis was abolished, showing that mechanical stretch may inhibit adipogenesis through Wnt signalling [39]. Normally, when MSCs are cultured in adipogenic medium, they express peroxisome proliferator-activated receptor γ (PPARγ) and adiponectin mRNA and protein, and accumulate intracellular lipids. However, when mechanical strain was applied to the MSCs for 6 h per day for 5 days, the expression of PPARγ and adiponectin was inhibited and the decrease seen in active and total β-catenin typically exhibited during adipogenesis was prevented. Mechanical strain was also thought to inactivate glycogen synthase kinase-3β, suggesting that stretch transmits anti-adipogenic signals *via* this pathway, by stimulating a durable β-catenin signal [40]. Another signalling pathway that is affected by mechanical stretch and has been implicated in the commitment of MSCs towards adipocytes is the BMP pathway, as it is thought that stretching causes a downregulation of BMP4 induced MSC adipogenesis. When MSCs were pretreated with BMP4 and then subjected to tensile stretch conditions (10% strain, 0.25 Hz, 120 min/day), it was found that the stretch supressed BMP4 induction of MSC adipogenesis and downregulated PPARγ, C/EBPα and aP2 adipogenic transcription markers, and lipid accumulation. Here, it was found that the cellular stretch did not affect BMP4-inducted activation in Smad or p38, as which this pathway would normally signal through Smad 1/5/8 and p38MAPK, but instead the tensile stretch caused significant ERK1/2 phosphorylation. When ERK signalling is blocked, the stretch suppression of BMP4-induced MSC adipogenesis was significantly deteriorated, suggesting that stretch suppresses BMP4-induced adipogenesis *via* upregulating ERK [41].

However, this may be specific to only BMP4-induced adipogenesis, as Li *et al.* (2015) reported that mechanical stretch did indeed upregulate levels of phosphorylated Smad2, along with PPARγ-2, adiponectin and C/EBPα. Here, the authors found that by pretreating MSCs with TGFβ1/Smad2 pathway antagonists suppressed this increase in Smad2 phosphorylation, whereas pretreatment with TGFβ1/Smad2 signalling agonists facilitated the inhibitory effect of stretch on the adipogenic differentiation markers, suggesting that the anti-adipogenic effects of stretch are mediated in some way by the activation of the TGFβ1/Smad2 signalling pathway [42].

### 2.5. Tenogenesis

Another way that human adult MSCs are thought to possess clinically useful tissue-regenerative properties is for the process of tendon tissue engineering, whereby they may be used to generate tenocytes for use in cell therapy. One way that this could be controlled and utilised is by using mechanical stretch techniques. At low-magnitude stretch, MSCs express osteogenesis differentiation marker genes, in agreement to the section above, but, interestingly, when stretched using high-magnitude stretch for long periods, the tendon and ligament related genes are instead upregulated. For example, after being stretched at 10% magnitude for 48 h, the expression of tenogenesis markers type I collagen, type III collagen, and tenascin-C are significantly increased [43]. This research is supported by findings from Morita *et al.* (2013), who demonstrated that a cyclic uniaxial stretch magnitude of 10% was the most efficient magnitude for inducing the differentiation of human BM-MSCs into tenocytes [44].

In the generation of tendon-or ligament-like tissue, MSCseeded 3D collagen gels are frequently utilised under static or dynamic tension, the latter of which leads to enhanced tendinous tissue development. Cyclic stretching has been found to be beneficial to this 3D system as it allows for the expression of the tendon marker scleraxis to be maintained, where it would have normally dropped off in expression, and there are vast changes in matrix deposition and remodelling activity under dynamic loading conditions. Furthermore, differential regulation of MMPs can be observed, with little change in collagen mRNA levels, giving insight to the mechanisms of tenogenesis following mechanical stimulation of MSCs [45].

The pathways that underlie how mechanical stretch impacts tenogenesis have also been investigated. Following mechanical stimulation, RhoA/ROCK and FAK have been found to regulate the mechanical-stretch induced realignment of human MSCs through cytoskeletal organization. Furthermore, after being subjected to mechanical stimulation, both RhoA/ROCK and cytoskeletal organization have been found to be essential in the phosphorylation of FAK at Tyr397. This phosphorylation process can be blocked by inhibiting either RhoA/ROCK, cytoskeletal organisation or FAK, implicating that these three are all vital components in the signalling network that senses mechanical stretch and then drives the tenogenic differentiation of human MSCs46. Moreover, when calcium signalling is disrupted in human MSCs, by blocking stretch activated calcium channels (SACC) with galolinium, before and whilst they are subjected cyclic uniaxial tensile stretching, almost all tenogenic differentiation marker expression enhancement and ECM production is lost, suggesting that SACC also act as a mechanosensor in the strain-induced model of human MSC tenogenesis [47].

### 2.6. Angiogenesis

The final major differentiation process which has been investigated in MSCs following mechanical stretch is regarding angiogenesis. Mechanical stretch has been found to increase the angiogenic capacity of MSCs *via* VEGFA induction, as well as increasing the survivability of MSCs under nutrient deprivation. The proposed mechanism by which both of these changes is thought to occur is *via* the activation and manipulation of NFκB; when subjected to stretch, there is an increase of nuclear localization of NFκB activity p65, which coincides with the increase in VEGFA expression and apoptosis resistance. When NFκB activity is inhibited, these pro-angiogenesis and anti-apoptosis functions are blocked, highlighting the significance of NFκB in the pro-angiogenic response following mechanical stretch48.

There are clearly a vast number of ways by which mechanical stretch can influence the many different differentiation pathways that MSCs are capable of, and the implications for increasing the therapeutic potential of MSCs are extremely exciting. Whether stretch is applied as a preconditioning technique or loading throughout implantation, for long or short durations and at high or low magnitudes and frequencies, there are clearly many ways it can be utilised, which will only become further understood in the years to come.

## 3. Other effects brought about by mechanical strain

In agreement with the above study, which demonstrated that stretch can have anti-apoptotic effects on human progenitor cells, work by Kearney *et al*. (2008) has also showed that mechanical strain can have wide reaching effects on the maintenance of MSCs; here the authors also reported that cyclic uniaxial stretch affects the apoptosis of MSCs. However, the authors instead reported that strains of 7.5% or greater, over a duration of three days, lead to an induction of apoptosis, with maximal apoptosis occurring at 10% of strain [49]. High levels of mechanical strain are thought to negatively impact MSCs as extreme stretch leads to oxygen free radical disequilibrium; when BM-MSCs from children were loaded with cyclic tensile strain, >12% magnitude stretch was found to enhance reactive oxygen species (ROS) synthesis, decrease the activity of superoxide dismutase and increase levels of malondialdehyde, in a time and magnitude dependent manner [50].

Another cellular function impacted by the mechanical microenvironment is intracellular calcium dynamics, as calcium oscillations can be effected by external mechanical cues. Prolonged mechanical stretch leads to intracellular calcium oscillations in human MSCs, as mediated by the cytoskeletal support, actomyosin contractility and phospholipase C (PLC) activity, showing another way by which the mechanical environment can regulate cellular functions [51].

**Fig. 2:**
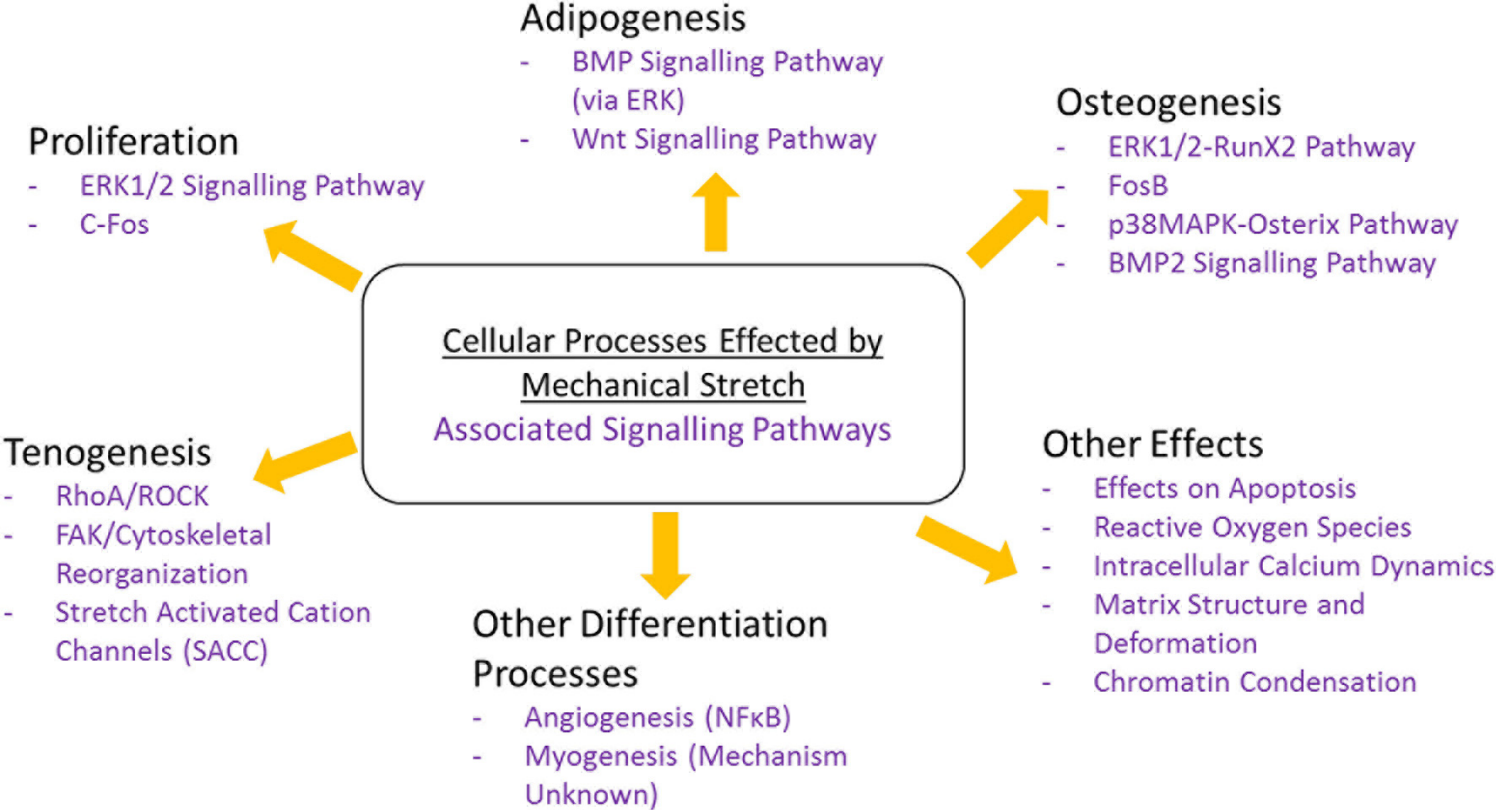
Summary of the common changes seen in adult stem cells following mechanical stretch.

Cyclic substrate deformation can also affect MSCs in terms of the matrix structure and formation of the cells. For example, equibiaxial cyclic strain (3%, 0.25 Hz) has been found to increase matrix mineralisation as well as inhibiting proliferation; here the strain was found to activate ERK1/2 and p38 MAPK pathways. When ERK1/2 was inhibited, this lead to an attenuation of calcium deposition, suggesting that strain-induced mineralisation was mediated by ERK1/2 signalling [52]. Furthermore, an increase in total collagen synthesis has been observed when MSCs were subjected to stretch conditions [53]. Research by Heo *et al.* (2015) found that short term dynamic loading causes increases in chromatin condensation, mediated by acto-myosin based cellular contractility and the activity of the histone-lysine N-methyltransferase EZH2. These changes stiffened the MSC nucleus, making it less deformable when subjected to stretch conditions. The authors identified ATP release and calcium signalling induced by mechanical stretch as the mediators of this condensation process. Following being subjected to stretch, it was also found that the cells retained a ‘mechanical memory’, whereby the cells exhibited higher amounts of chromatin condensation that persisted for longer times, when subjected to increasing numbers of loading events and strain levels, which may be a mechanism by which the MSCs sensitize themselves to future loading events [54]. Collectively, these studies emphasise just how far reaching the effects of mechanical stretch can be on MSC physiology and maintenance, and show how tensile loading can affect not only cellular differentiation, but also affect apoptosis, ROS, calcium oscillations and matrix mineralisation. A summary of the effects on cellular processes brought about by mechanical strain can be found below (Fig. 2).

## 4. Mechanical stretch and biomaterials: physiological loading and the role of the circadian rhythm

Unsurprisingly, the effects that mechanical loading can have on a biomaterial seeded and optimised with human MSCs are widespread and well documented. There are an enumerable amount of new materials being created and optimised constantly, and each of these may be impacted and improved by subjecting the material, and cells within, to mechanical load, or by preconditioning the cells with mechanical strain. For example, stem cell-collagen sponge constructs increase in stiffness following mechanical stimulation. Two weeks of *in vitro* mechanical stimulation was here found to increase collagen type I and type III gene expression and an increase in linear stiffness and linear modulus [55]. Long term dynamic compression of MSC-seeded hydrogel constructs initiated after chondrogenesis induction has also been found to enhance matrix distribution and the mechanical properties of MSC-seeded constructs [56], showing again how mechanically stimulating cells in constructs can improve function. Electrospun fibres, which are advantageous in connective tissue regeneration in terms of their durability, deformation capacity and effects on cell adhesion, orientation and gene expression, experience an increase in cell alignment when subjected to cyclic loads, suggesting that cell morphology within a construct is also influenced by the mechanical environment [57].

However, this is not the only physiological process that is effected by the mechanoenvironment of MSCs following implantation into biomaterials. Research by Mengatto *et al.* (2011) explored osseointegration implant failure, to see what impacted the establishment of dental and orthopaedic implant association with bone tissue. To do this, the authors used a vitamin D deficiency model of implant failure in rats and evaluated changes in gene expression using whole genome microarray analyses. KEGG analysis was utilised and it was found that 103 genes were significantly modulated by implant placement and vitamin D deficiency, with the highest z-scores assigned to components of the circadian rhythm pathway, including neuronal PAS domain 2 (NPAS2) and period homolog 2 (Per2). Furthermore, NPAS2 and Aryl hydrocarbon receptor nuclear translocator-like (ARNTL/Bmal1) were found to be upregulated, and Per2 showed a complementary expression pattern following the vitamin D model. This study suggests that the circadian rhythm may have a key role in the establishment of osseointegration under vitamin D regulation [58].

**Fig. 3:**
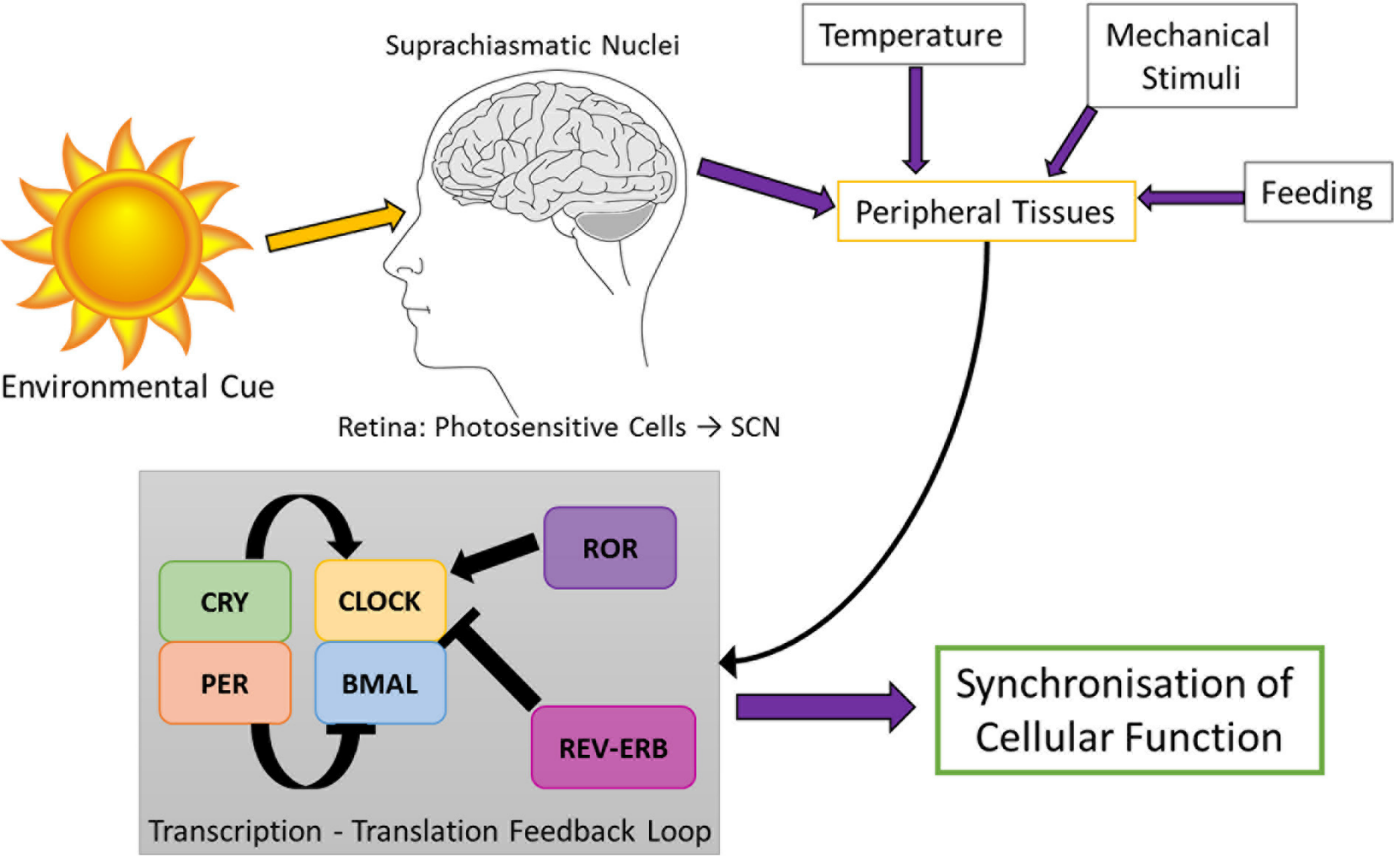
Summary Schematic of the Circadian Rhythm. Light enters the brain through the retina and is relayed from the photosensitive cells to the SCN. The SCN then signals to a number of peripheral tissues and cells all over the body where it feeds into the molecular clock auto-regulatory feedback loop, which act on target genes and lead to the synchronisation of cellular function.

The circadian rhythms are important evolutionarily conserved cellular mechanisms which are a subset of biological rhythms, they have a period, *i.e.* the time taken to complete one cycle, of approximately 24 hours. The foundation of circadian rhythmicity research is often dated back to the work done by Colin Pittendrigh and Jurgen Aschoff. These pioneers are thought to have defined the basis of circadian entrainment. Pittendrigh (1960) showed that deviation from the 24-hour cycle provides a mechanism for alignment for the internal time-keeping system, allowing the rhythm to be “reset” where necessary [59]. The molecular mechanism used to generate self-sustained circadian rhythms rely on a network of auto-regulatory feedback loops of transcription and translation to drive circadian expression patterns of the core clock components [60]. In mammals, this is carried out by the primary feedback loop by the basic-helix-loop-helix transcription factors CLOCK and BMAL, which form the positive arm of the molecular clock. When these two proteins heterodimerise, they are able to bind to cis-regulatory enhancer sequences called E-boxes on target gene promoters, and so initiate transcription [61, 62]. Target genes include Period (Per1, Per2 and Per3) and Cryptochrome (Cry1 and Cry2), which themselves heterodimerise and translocate to the nucleus to affect gene expression. However, unlike the CLOCK:BMAL complex, they have a negative feedback effect, and repress their own transcription by inhibiting the CLOCK:BMAL complex [63, 64]. The CLOCK:BMAL heterodimers induce a stabilising regulatory loop by activating the transcription of retinoic acid-related orphan nuclear receptors, REV-ERB and ROR. These bind to retinoic acid-related orphan receptor response elements (ROREs), which are present in Bmal gene promoter. REV-ERBs repress transcription of Bmal, whereas RORs activate it [65]. These auto-regulatory loops constitute a molecular clock machinery and take approximately 24 hours to complete a cycle (Fig. 3).

Similarly to Mengatto *et* al.’s work, Hassan *et al.* (2017) also found that the circadian rhythm may influence the fate of certain biomaterials. In this instance, the circadian rhythm of BM-MSCs was found to be induced by Titanium (Ti) -based biomaterials with complex surface modifications (Ti biomaterials). When human MSCs were cultured on Ti biomaterials, it was found that Per1 expression was suppressed, whereas NPAS1 was upregulated. BM-MSCs were then harvested Npas2 knockout mice, it was found that this did not rescue the Ti biomaterial-induced reduction of Per1 expression, and did not affect Per2, Per3, Bmal1 or Clock expression, suggesting that the Ti biomaterial-induced increase in NPAS1 expression was independent of the changes in circadian component expression. The authors also found that vitamin D supplementation significantly increased Per1 expression in BM-MSCs66. Taken together, both of these studies suggest that the circadian rhythm of BM-MSCs may influence the integration of Ti biomaterials into bone, and therefore should be taken into account in future biomaterial research.

## 5. Using the cellular mechanoenvironment to control the circadian rhythm of adult progenitor cells

The idea that mechanical stimulation could be used to direct and control the circadian rhythm was first investigated by Simoni *et al.* (2014). Drosophila melanogaster were here exposed to 12 hour: 12 hour cycles of vibration and silence, and it was found that this was sufficient to entrain and synchronise their behaviour and daily locomotor activity. In order for this to occur, the Drosophila required both a functional clock and functional chordotonal organs, as the mechanosensory input pathway to the fly’s circadian clock required signalling from the chordotonal organs in order to synchronise their circadian rhythm [67]. This exciting research then led to other findings from our own group; it was published that different human adult progenitor cells have peripheral circadian rhythms of their own and the cells are capable of being synchronised on a molecular level by a number of different means, including both by chemical and mechanical stimulation. Human progenitor cells derived from tooth dental pulp, subcutaneous adipose and bone marrow were all exposed to synchronisation by dexamethasone, a synthetic glucocorticoid, and it was found that the more mature ADSCs and BM-MSCs could be readily synchronised in response to this treatment. The more primitive DPSCs, however, were less able to respond to this form of entrainment, which could again be due to their unique developmental origin. Next, the different progenitor cells were exposed to cyclic tensile stretch of 12 hour: 12 hour cycles of stretch and relaxation (6.66% stretch, 1 Hz, 12 h/day) for three days. This was found to be sufficient to entrain the progenitor cells of different tissue sources, including the DPSCs, showing the diverse functionality that mechanical stretch has to offer [68]. This offers a novel, nonintrusive methodology by which the circadian rhythm of progenitor cells can be poised and made ready for implantation.

The circadian rhythm of adult progenitor cells has also been investigated in response to the mechano-chemical stiffness of the cellular microenvironment, as circadian clock genes have been previously linked to mammary progenitor cell function. Notably, it has recently been published that the mechanical environment of the epithelial progenitor cell niche within mammary tissue controls the amplitude of molecular clock oscillations, which can be altered upon environmental and genetic clock disruption. Moreover, cell-matrix interactions do indeed play a key role in regulating circadian biology, and tissue stiffening is now thought to suppress the mammary circadian clock *in vivo,* where the mammary clock may be controlled by the periductal extracellular matrix. Mechanistically, vinculin, a tension sensing cell-matrxc adhesion molecule, and the Rho/ROCK pathway, which transmits extracellular matrix stiffness signals into cells, are both thought to influence the regulation of the circadian clock. Furthermore, by disrupting the circadian clock of mammary progenitor cells, this leads to disruptions in the self-renewal capacity of the mammary epithelia, again highlighting the key roles of the mechanoenvironment and the circadian clocks of the epithelial stem cell niche in progenitor cell function [69].

## 6. Conclusion

In this review, we have explored the broad ranging effects that mechanical strain can have on adult progenitor cell activation and maintenance. It has been shown that tensile stretch has the capacity to influence not only adult progenitor cell proliferation and differentiation into a number of lineages relevant to cell based tissue engineering and regenerative medicine today, but they can also have profound effects on progenitor cell homeostasis, the optimisation of biomaterials and even the circadian rhythm. Now is an extremely exciting time for mechano-biology, which will only continue to grow in terms of the knowledge base, along with realising the potential impacts and significance that have already been experimentally evidenced with respect to controlling and defining progenitors cells, the implications for future regenerative medicine and tissue engineering are extremely positive and optimistic looking.

## Conflicts of interest

The authors declare no conflict of interest.
